# Orthodontic Pain and Dietary Impact Considering Age Groups: A Comparative Study

**DOI:** 10.3390/jcm13041069

**Published:** 2024-02-14

**Authors:** Bianca-Maria Negruțiu, Luminița Ligia Vaida, Claudia Judea-Pusta, Cristian Romanec, Abel Emanuel Moca, Cristina Paula Costea, Claudia-Elena Staniș, Marius Rus

**Affiliations:** 1 Department of Dentistry, Faculty of Medicine and Pharmacy, University of Oradea, 410073 Oradea, Romania; 2 Department of Morphological Disciplines, Faculty of Medicine and Pharmacy, University of Oradea, 410073 Oradea, Romania; 3 Department of Orthodontics and Dentofacial Orthopedics, Faculty of Dentistry, “Grigore T. Popa” University of Medicine and Pharmacy, 700115 Iași, Romania; 4 Faculty of Medicine and Pharmacy, University of Oradea, 410073 Oradea, Romania; stanis.claudia@yahoo.com; 5 Department of Medical Disciplines, Faculty of Medicine and Pharmacy, University of Oradea, 410073 Oradea, Romania; rusmariusr@yahoo.com

**Keywords:** orthodontics, oral healthcare, pain, weight loss, interceptive orthodontics

## Abstract

**(1) Background**: orthodontic treatment can frequently be associated with discomfort and pain, a significant factor contributing to treatment discontinuation. **(2) Methods**: This study, conducted on 160 orthodontic patients across different age groups, aimed to explore the influence of age on patients’ responses to treatment, particularly regarding changes in dietary patterns and weight loss. The patients were categorized into three age groups and assessed through a questionnaire about pain perception, pain latency, dietary changes, and weight loss associated with orthodontic appliances. **(3) Results**: Younger patients (6–12 years) reported lower pain levels, shorter pain latency and fewer alterations in dietary habits compared to adults (over 18 years). Females over 18 represented a significant portion of the sample, suggesting a self-driven inclination towards orthodontic treatment for aesthetic reasons. Fixed orthodontic appliances induced more significant pain than removable ones. Adults experienced more changes in dietary habits and weight loss than younger individuals. **(4) Conclusions**: the results provide valuable insights for orthodontic practitioners aiming to mitigate adverse effects and improve overall patient experience during treatment.

## 1. Introduction

Orthodontic treatment often triggers discomfort or pain, which is considered one of the most important reasons to discontinue treatment [1]. Other frequent complaints include oral ulcerations, tongue soreness and functional limitations [2]. These can influence patients’ quality of life, oral health, masticatory function, and eating habits [3]. Moreover, social anxiety, fear of being rejected by peers, and conflicts with adults can occur before and after orthodontic treatment [4].

All these drawbacks can lead to orthodontic patients complaining about food restrictions, leading to weight loss during orthodontic treatment, thus negatively influencing patient compliance to treatment [5]. Also, they tend to prefer soft food in order to minimize the discomfort or pain caused by the appliance, which can have a direct consequence on the nutritional value and quality of the food they ingest [6,7].

Moreover, due to the fact that most of the patients undergo orthodontic treatment during their adolescence, when nutritional needs are critical, orthodontists should try not to affect patients’ diets, as this can interfere with their patients’ growth and development [8,9]. Since 2016, Sandeep et al. have emphasized that orthodontic patients must pay attention towards their nutrients and consume foods that are rich in nutritional values in order to maintain a good overall health [10].

The type of pain triggered by an orthodontic fixed appliance (brackets, arch wires, auxiliary springs and separators) shows large individual variations and is influenced by several factors, such as age, gender, the magnitude of force applied, emotional state and stress, as well as previous pain experiences [11,12,13]. According to Johal, Fleming and Al Jawad (2014), orthodontic pain starts 4 h after adjusting the orthodontic appliance, reaches the peak between 12 h and 3 days, and decreases gradually for up to 7 days after the adjustment [14].

However, according to Feldmann, List and Bondemark (2011), after 4–6 weeks from adjusting the fixed orthodontic appliance, the reduced masticatory ability returns to its baseline [15]. The aim of this paper was to determine whether age influences patients’ response to orthodontic treatment, thus leading to changes in dietary patterns and weight loss.

## 2. Materials and Methods

### 2.1. Sample Selection

This study was conducted in June 2022 on a group of 160 orthodontic patients, in accordance with the World Medical Association (WMA) Declaration of Helsinki–Ethical Principles for Medical Research Involving Human Subjects. All patients included in this study gave their consent. For the under-aged patients, a paternal consent was obtained.

The group of patients was divided into 3 age categories: 6–12 years (26 patients, 18 females and 8 males), 12–18 years (82 patients, 54 females and 28 males), over 18 years (52 patients, 48 females and 4 males).

The research consisted of applying a questionnaire based on 7 single-choice questions. The questions referred to the age category of the respondent (6–12 years, 12–18 years and over 18 years), gender, type of pain that the patient experienced after activating the orthodontic appliance (slight discomfort, mild pain, moderate pain, severe pain), pain latency (no pain, 5 min, few hours, one day, one week), any change in dietary habits, weight loss associated with orthodontic appliance and type of pain correlated with the type of orthodontic appliance used (fixed or removable).

Orthodontic pain was assessed on a visual analogue scale of 0–10. The pain scale was then categorized into slight discomfort (1–3), mild pain (4–5), moderate pain (6–7) and severe pain (8–10).

The main inclusion criteria in this study were to undergo an orthodontic treatment (fixed or removable) for at least one month, non-syndromic patients and/or with craniofacial deformities, cleft lip or palate, and no general disease history.

### 2.2. Statistical Analysis

IBM SPSS software, version 20 (IBM, Chicago, IL, USA) was used for statistical analysis. The Shapiro–Wilk Test was used to determine the distribution of quantitative data, which were expressed as mean values with standard deviations (or medians with inter-percentile intervals, depending on the distribution), while categorical variables were expressed in absolute or percentage form. The Mann–Whitney U or Kruskal–Wallis H Test was used to evaluate the independent quantitative variables because their distribution was nonparametric. Spearman’s Rho Correlation Coefficient was used to show the existent correlations, while Fisher’s Exact Test or Pearson’s Chi-Square Test were used to determine the qualitative data. Each correlation was proven using the Pearson Correlation Coefficient. Z-tests with Bonferroni corrections were performed in order to express further details of the results obtained after testing qualitative data. Dunn–Bonferroni tests were performed post hoc in order to elaborate the results obtained after testing the independent quantitative variables.

### 2.3. Ethical Considerations

The study was conducted in accordance with the 1964 Declaration of Helsinki and its later amendments, and it was approved by the Research Ethics Committee of the University of Oradea (No. 12/06.05.2022). Informed consent was obtained from each respondent or legal tutor.

## 3. Results

Data shown in Table 1 show the distribution of patients in regard to age category and gender. The differences between groups were tested using Fisher’s Exact Test, the results proving the existence of statistically significant differences (*p* = 0.002). The slight negative correlation (*p* = 0.007, R= −0.223) and Z-tests with Bonferroni corrections show that, among patients aged 12–18 years, the share of males (70%) is significantly higher than the share of females (45%). Also, among patients over 18, the share of males (10%) is significantly lower than the share of females (40%).

**Figure 1:**
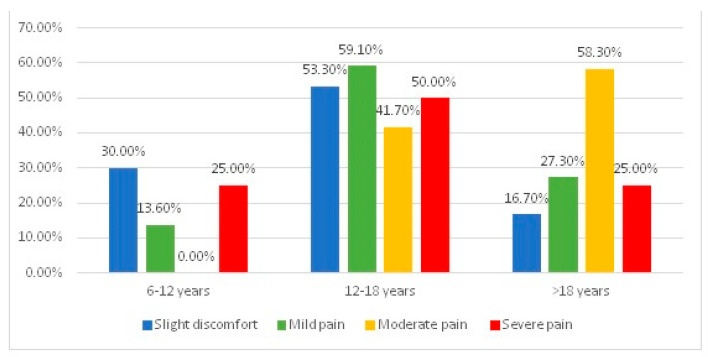
The distribution of patients regarding age category and type of pain.

Figure 1 represents the distribution of patients according to age category and the type of pain the orthodontic appliance generates after activation. The differences between groups were tested using Fisher’s Exact Test, the results proving the existence of statistically significant differences (*p* < 0.001). Moderate positive correlation (*p* < 0.001, R = 0.348) and Z-tests with Bonferroni corrections show that, among patients aged 6–12, the proportion of those who perceived moderate pain after the activation of the orthodontic appliance was significantly lower (0%) than the share of those who perceived another type of pain. Also, among patients over 18, the share of those who perceived severe pain (58.3%) was significantly higher than the share of those who perceived mild pain (27.3%) or only slight discomfort (16.7%) after the activation of the orthodontic appliance.

**Figure 2:**
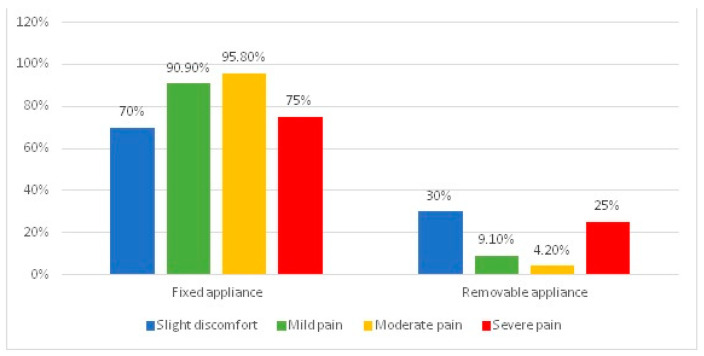
The distribution of patients according to the type of orthodontic appliance they wear and the degree of pain it triggers after activation.

For a better understanding of these results, Figure 2 represents the distribution of patients according to the type of orthodontic appliance they wear and the degree of pain it triggers after activation. The differences between groups were tested using Fisher’s Exact Test, the results proving the existence of statistically significant differences (*p* = 0.002). The slight negative correlation (*p* = 0.004, R = −0.229) and Z-tests with Bonferroni corrections show that, among patients with fixed orthodontic appliances, the share of those with moderate pain (95.8%) was significantly higher than the share of those with only a slight discomfort (70%), while among patients with removable orthodontic appliances, the proportion of those with moderate pain (4.2%) was significantly lower than the proportion of those with only slight discomfort (30%).

Table 2 shows the distribution of patients according to age and pain latency. The differences between groups were tested using Fisher’s Exact Test, the results proving the existence of statistically significant differences (*p* < 0.001). The high degree positive correlation (*p* < 0.001, R = 0.564) and Z-tests with Bonferroni corrections show that, among patients aged 6–12, the highest proportion were those who had a pain latency of up to 5 min (41.2%), significantly higher compared to those with a latency of pain of several hours (12.5%) or a day (0%). Also, in the same age category, the share of those who did not experience pain (28.6%) was significantly higher than those with a latency of pain of one day (0%). Among patients older than 18, the highest share was of those with a latency of pain of one day (65.2%) significantly higher compared to those with a latency of several hours (31.2%), 5 min (0%) or no pain (0%). Also, the share of those with a latency of 5 min (0%) was significantly lower than the share of those with a latency of pain of a few hours (31.2%) or a week (100%).

**Figure 3:**
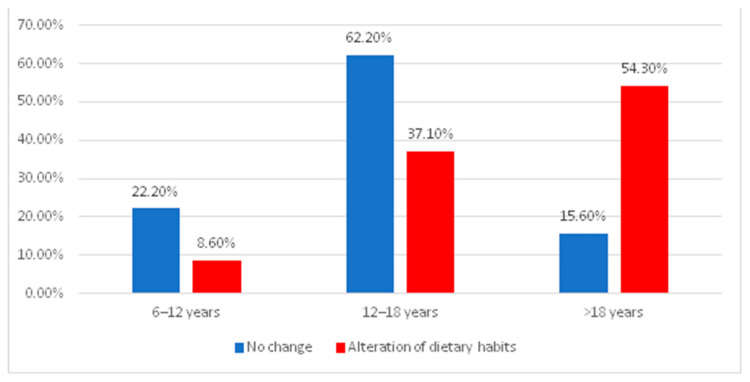
The distribution of patients according to age and the existence of changing dietary habits.

The data in Figure 3 represent the distribution of patients according to age and the existence of changing dietary habits after the activation of the orthodontic appliance. The differences between groups were tested using the Pearson Chi-Square Test, with the results proving the existence of statistically significant differences (*p* < 0.001). Moderate positive correlation (*p* < 0.001, R = 0.383) and Z-tests with Bonferroni corrections show that, between patients aged 6–12 and those aged 12–18, the proportion of those with unchanged eating habits (22.2%/62.2%) was significantly higher than the share of those with altered dietary habits (8.6%/37.1%), while among patients older than 18, the share of those with impaired nutrition (54.3%) was significantly higher than the share of those with unaffected nutrition habits (15.6%).

Moreover, Table 3 shows the distribution of patients considering age and weight loss after the activation of the orthodontic appliance. The differences between groups were tested using Fisher’s Exact Test, with the results proving the existence of statistically significant differences (*p* = 0.001). The mild positive correlation (*p* < 0.001, R = 0.285) and the Z-tests with Bonferroni corrections show that, among patients aged 6–12, the share of those with no weight loss was significantly higher (21.4%) than those with weight loss (4.2%), while among patients older than 18, the share of those with weight loss (50%) was significantly higher than those with no weight loss (25%).

## 4. Discussion

The procedure of aligning teeth offers both aesthetical and functional advantages for the patient, thus improving their self-esteem and confidence [16]. The means of achieving the goals (placing separators, initial arch wires, monthly adjustments) involves discomfort, pain, changing eating patterns, or even weight loss for the patient.

The sample of subjects we analyzed (160 orthodontic patients divided into three age categories) shows a significantly higher share of males in the category of patients aged 12–18 years. This result can be explained by the fact that male adolescents are frequently taken to the orthodontist by parents concerned about their children’s oral health. Moreover, a significantly higher share of females aged over 18 who undergo orthodontic treatment can be noticed in the analyzed sample. This result could be due to women considering their smile to be the most important aspect of facial attractiveness compared to men, making them more careful with their physical appearance, and thus deciding on their own to undergo orthodontic treatment [17].

Comparing the age categories 6–12 and over 18, it is notable that younger patients experience lower levels of pain compared to adult patients. This can be explained by the fact that more adult patients are treated using fixed orthodontic appliances compared to patients aged 6–12, who mostly undergo removable orthodontic treatments. These results are similar to those obtained by Baseer et al. (2021), who stated that fixed orthodontic patients reported significantly higher pain intensity than removable orthodontic patients [18]. Similar results were found by Sultan H. et al. (2023), who showed that patients aged 21–36 experienced more pain during mastication after placing separators for fixed appliances than the group aged 9–20 years [19].

Some authors found no correlation between pain and age [20], whilst other authors, such as Bergius et al. (2000) and Jones (1984), reported higher levels of pain in older subjects [12,21]. Bergius et al. (2000) observed a linear negative correlation between age and general pain until the age of 25; however, in orthodontics the relationship was not linear, and the age category with the lowest pain level seemed to be 13–16 years [12].

Moreover, complex central interactions between specific neurotransmitters, such as serotonin and opiates, as well as ovarian steroids, may influence the response to orthodontic treatment, leading to exaggerated responses to painful stimulus [22]. Myers et al. (2006) showed that pain perception among boys and girls changes significantly after puberty, with girls experiencing greater pain due to hormone level fluctuations during the menstrual cycle, which lowers pain thresholds [23]. Psychological factors represented by depression, anxiety, or low self-esteem can also be associated with increased clinical pain responses to orthodontic treatment [11].

Patients who underwent fixed orthodontic treatment experienced mostly moderate pain compared to patients who underwent removable orthodontic treatment, who claimed only slight discomfort. This may be due to the fact that all the patients who underwent fixed orthodontic treatment had a high incidence of mucosal sores on the lips, tongue and cheeks, which decreased pain thresholds [18,24]. Our results are similar to those reported by Wiedel et al. (2015) [25], who stated that fixed orthodontic patients had higher pain intensity than removable orthodontic patients. However, this is in contrast to the study published by Alajmi et al. (2020) [26], who underlined that both fixed and removable orthodontic patients experienced the same level of pain intensity.

Patients aged 6–12 claimed 5 min pain latency (or even no pain latency) after the adjustment of the orthodontic appliance compared to patients over 18, who experienced a pain latency of up to one day. This result can be explained by the fact that patients over 18 often undergo orthodontic fix treatment, not being able to remove the appliance, whilst patients aged 6–12 undergo removable orthodontic treatment, thus being able to discontinue the treatment by removing the appliance.

Since 1971, Soltis et al. have reported that orthodontic procedures reduces the proprioceptive and discriminating abilities of the patients for up to 4 days [27]. Moreover, orthodontic force application determines both an immediate painful response due to compression and a delayed painful response due to hyperalgaesia of the periodontal ligament. Thus, the periodontal ligament is sensitive to released algogens, such as histamine, bradykinin, prostaglandins, and serotonin [28,29]. According to Bergius et al. (2000), the painful response continues with neurogenic inflammation, osteoblastic and osteoclastic activity, as well as periodontal vasodilation [12].

Pre-adolescent (6–12) and adolescent (12–18) patients claimed to have no changes in eating patterns compared to adult patients (over 18 years), who did experience alterations in their dietary habits. This result can be explained by the fact that adult patients claim a higher level of pain during orthodontic treatment, thus reducing their intake of food and the pleasure of eating, eventually leading to a decrease in weight. Similar to our results, Johal A. et al. (2013) stated that, during the first three months of orthodontic treatment, no significant effect on dietary intake or behavior, body mass index and fat percentage was found [30].

However, Ozdemir et al. (2021) [31] concluded that, in adolescents who underwent orthodontic treatment, vitamin C, vitamin E and fiber intake decreased significantly, especially in the first week of treatment, without reaching their initial levels by the end of the 12th week of treatment.

Our results are similar to Baseer et al. (2021) [18], who demonstrated that removable orthodontic patients had significantly lower food impactions than fixed orthodontic appliances due to the fact that removable appliances can be removed during eating. However, slight discomfort can be experienced during food intake even with removable orthodontic patients due to tooth sensitivity during orthodontic movement [32]. Studies conducted in 2018 and 2013 showed that over half the patients reported difficulty in chewing hard food and felt more comfortable with soft food and liquids [33,34]. Similar to our results, Anjwa et al. (2018) stated that patients who experienced discomfort when eating reported weight loss after orthodontic treatment [33]. Also, Al Jawad et al. (2012) emphasized that orthodontic patients experienced changes in their diet but felt that their eating habits became healthier during the orthodontic treatment [9]. Similar results were reported by Aljohani et al. (2020), who emphasized that patients’ oral health behavior was improved during and after orthodontic treatment [35].

Some limitations of the present study need to be considered. One of them refers to the fact that the preadolescent group underwent both fixed and removable treatment whilst the adolescent and adult group underwent exclusively fixed orthodontic treatment. Removable appliances did not include invisible aligner therapy. Other limitations refer to the fact that the results were collected from a questionnaire in which the answers can be influenced by the psychological state of the patient at that particular moment. Also, weight loss was not determined by using the body mass index, but by trusting the validity of the patients’ responses.

## 5. Conclusions

The type of orthodontic treatment (fixed or removable) and the age of the patient while undergoing orthodontic treatment influenced patients’ response to treatment. The younger the patient, the less negative responses to orthodontic treatment they experienced (pain, changes of the dietary patterns, weight loss). The results of this study should be considered by orthodontists in order to help alleviate patients from the beginning pain, discomfort and anxiety determined by an orthodontic treatment, thus raising the levels of competence and professionalism of the orthodontist. As a result, clinicians will have a better understanding of their patients’ quality of life and their expectations about the orthodontic treatment.

## Figures and Tables

**Table 1:** The distribution of patients regarding age and gender.

Age Category/Gender	Female		Male		*p* *
	No.	%	No.	%	
6–12 years	18	15%	8	20%	0.002 0.007, R = −0.223 **
12–18 years	54	45%	28	70%	
>18 years	48	40%	4	10%	

* Fisher’s Exact Test, ** Pearson Correlation Coefficient.

**Table 2:** The distribution of patients according to age and pain latency.

Age Category/Pain Latency	No Pain		5 Min		Few Hours		1 Day		1 Week		*p* *
	Nr.	%	Nr.	%	Nr.	%	Nr.	%	Nr.	%	
6–12 years	4	28.6%	14	41.2%	8	12.5%	0	0%	0	0%	<0.001 <0.001, R = 0.564 **
12–18 years	10	71.4%	20	58.8%	36	58.2%	16	34.8%	0	0%	
>18 years	0	0%	0	0%	20	31.2%	30	65.2%	2	100%	

* Fisher’s Exact Test, ** Pearson Correlation Coefficient.

**Table 3:** The distribution of patients considering age and weight loss after the activation of the orthodontic appliance.

Age Category/Weight Loss	No Weight Loss		With Weight Loss		*p* *
	No.	%	No.	%	
6–12 years	24	21.4%	2	4.2%	0.001 <0.001, R= 0.285 **
12–18 years	60	53.6%	22	45.8%	
>18 years	28	25%	24	50%	

* Fisher’s Exact Test, ** Pearson Correlation Coefficient.

## Data Availability

The data presented in this study are available on request from the corresponding authors. The data are not publicly available due to privacy reasons.
